# Association between the atherogenic index of plasma and adverse long-term prognosis in patients diagnosed with chronic coronary syndrome

**DOI:** 10.1186/s12933-023-01989-z

**Published:** 2023-09-21

**Authors:** Jiasuer Alifu, Lanqing Xiang, Wen Zhang, Penglong Qi, Huiying Chen, Lu Liu, Guoqing Yin, Abdul-Quddus Mohammed, Xian Lv, Tingting Shi, Fuad A. Abdu, Wenliang Che

**Affiliations:** 1 Department of Cardiology, Shanghai Tenth People’s Hospital, Tongji University School of Medicine, 301 Yanchang Road, Shanghai, 200072 China; 2 Department of Cardiology, Clinical Medical College of Shanghai Tenth People’s Hospital, Nanjing Medical University, Shanghai, China; 3 Department of Cardiology, Shanghai Tenth People’s Hospital Chongming branch, Shanghai, China

**Keywords:** Chronic coronary syndromes, Atherogenic index of plasma, Prediction, Clinical outcomes

## Abstract

**Background:**

The Atherogenic Index of Plasma (AIP) is a newly identified biomarker associated with lipid metabolism, demonstrating significant prognostic capabilities in individuals diagnosed with cardiovascular disease. However, its impact within the context of chronic coronary syndromes (CCS) remains unexplored. Thus, the present investigation sought to examine the potential association between AIP levels and long-term clinical outcomes in patients diagnosed with CCS.

**Methods:**

A total of 404 patients diagnosed with CCS and who underwent coronary angiography were included in this study. The AIP index was calculated as log (triglycerides / high-density lipoprotein-cholesterol). The patients were categorized into four groups based on their AIP values: Q1 (< -0.064), Q2 (-0.064 to 0.130), Q3 (0.130 to 0.328), and Q4 (> 0.328). The occurrence of major adverse cardiovascular events (MACE) was monitored during the follow-up period for all patients. Cox regression analysis and Kaplan-Meier curve analysis were employed to examine the relationship between AIP and MACE. Furthermore, ROC analysis was utilized to determine the optimal cut-off value of AIP for predicting clinical MACE.

**Results:**

During the median 35 months of follow-up, a total of 88 patients experienced MACE. Notably, the group of patients with higher AIP values (Q4 group) exhibited a significantly higher incidence of MACE compared to those with lower AIP values (Q1, Q2, and Q3 groups) (31.7% vs. 16.8%, 15.7%, and 23.0% respectively; P = 0.023). The Kaplan-Meier curves illustrated those patients in the Q4 group had the highest risk of MACE relative to patients in the other groups (log-rank P = 0.014). Furthermore, the multivariate Cox regression analysis demonstrated that individuals in the Q4 group had a 7.892-fold increased risk of MACE compared to those in the Q1 group (adjusted HR, 7.892; 95% CI 1.818–34.269; P = 0.006). Additionally, the ROC curve analysis revealed an optimal AIP cut-off value of 0.24 for predicting clinical MACE in patients with CCS.

**Conclusion:**

Our data indicate, for the first time, that AIP is independently associated with poor long-term prognosis in patients suffering from CCS. The optimal AIP cut-off value for predicting clinical MACE among CCS patients was 0.24.

**Supplementary Information:**

The online version contains supplementary material available at 10.1186/s12933-023-01989-z.

## Introduction

Coronary artery disease (CAD) represents a significant public health burden, impacting a substantial global population of 244.11 million individuals and exhibiting the highest fatality rate among all cardiovascular conditions [1–3]. Given the dynamic nature of the CAD process, leading to diverse clinical manifestations, it is conveniently classified into acute coronary syndromes (ACS) and chronic coronary syndromes (CCS), which constitute a substantial proportion of CAD cases [4]. According to the 2016 data released by the American College of Cardiovascular Diseases, it is projected that by the year 2030, approximately 18% of adults will be affected by CCS, thereby posing a grave concern to the overall well-being of individuals [5]. Therefore, identifying reliable biomarkers associated with CCS is crucial for early detection, risk assessment, and targeted interventions to prevent or manage the disease. Nonetheless, there exists a scarcity of studies focusing on this specific patient group that utilizes clinically valuable indicators to prognosticate adverse clinical outcomes related to CCS.

The Atherogenic Index of Plasma (AIP) represents a logarithmically transformed quotient of plasma triglycerides (TG) to high-density lipoprotein-cholesterol (HDL-C) (Log (TG/HDL-C)) [6, 7]. It has demonstrated significant associations with HDL-C, low-density lipoprotein-cholesterol (LDL-C), and very low-density lipoprotein (VLDL) particle sizes, thereby establishing its predictive capability for cardiovascular disease (CVD) risk [8, 9]. Numerous studies have investigated the association between AIP and various CVD, including ACS, atherosclerosis, and ST-segment elevation myocardial infarction (STEMI) after primary percutaneous coronary intervention (PCI) [10–12]. Zheng et al. demonstrated the utility of AIP in prognosticating outcomes among non-diabetic patients with CAD who had undergone PCI over a two-year follow-up period [13]. Elevated AIP values have been documented in patients with ACS and have been employed as an indicator to assess the extent of lipid-driven inflammation within this population [10]. In STEMI patients following PCI, the AIP exhibits superior predictive capabilities in comparison to individual measurements of TG and HDL-C levels [12]. However, despite the extensive studies on the relationship between AIP and CVD, there is no data specifically examining the association between AIP and patients with CCS. Understanding the potential role of AIP in the pathogenesis and progression of CCS could provide valuable insights into its underlying mechanisms and help identify novel therapeutic targets.

Consequently, the primary objective of this study was to investigate the association between AIP and the likelihood of experiencing major adverse cardiovascular events (MACE) in patients with CCS.

## Materials and methods

### Study design and population

The present study is a single-center retrospective observational study that was conducted at Shanghai Tenth People’s Hospital from June 2015 to June 2019. A consecutive cohort of 404 subjects admitted for CCS and undergoing coronary angiography (CAG) were recruited for the study. The patients diagnosed with suspected or established CCS according to the 2019 European Society of Cardiology (ESC) guidelines [4] and aged over 18 years old were enrolled in this study. The major exclusion criteria were consisting of the following items: (1) recent occurrence of myocardial infarction (MI) within 7 days; (2) post coronary artery bypass graft surgery (CABG); (3) severe hepatic or renal insufficiency; (4) malignancy; (5) left ventricular ejection fraction (LVEF) below 35%; (6) presence of other major diseases significantly affecting long-term survival; (7) instances of patient loss to follow-up or incomplete AIP data. Our study was carried out in accordance with the Helsinki Declaration and was approved by the ethical review board of Shanghai Tenth People’s Hospital (ethical number: SHSY-IEC-5.0/23K92/P01). Each participating patient in this study recruited written informed consent.

### Data collection and definitions

Baseline demographic data, encompassing variables such as age, gender, height, weight, body mass index (BMI), heart rate, and blood pressure, along with pertinent clinical information, including past medical history (e.g., hypertension, diabetes, hyperlipidemia, atrial fibrillation, smoking, chronic kidney disease (CKD), heart failure, and stroke), coronary angiographic findings, echocardiography parameters, and medication history, were retrospectively acquired from the medical records of all study participants. Laboratory parameters were obtained after an overnight fast via venous blood samples on admission to measure fasting blood glucose (FBG), hemoglobin A1c (HbA1c), total cholesterol (TC), TG, LDL-C, serum creatinine (SCr), C-reactive protein (CRP), alanine aminotransferase (ALT), aspartate aminotransferase (AST), estimated glomerular filtration rate (eGFR), and hemoglobin (Hb) levels. Blood glucose, TC, LDL-C, HDL-C, and TG were analyzed using Abbott Laboratories (Chicago, IL, USA). A diabetes diagnosis is established on the following criteria: (1) FBG ≥ 7.0 mmol/l (≥ 126 mg/dl); (2) Random plasma glucose ≥ 11.1 mmol/l (≥ 200 mg/dl); (3) OGTT glucose level ≥ 11.1 mmol/l (200 mg/dl); and (4) HbA1c ≥ 6.5%. The eGFR was calculated using the Modification of Diet in Renal Disease Study (MDRD) GFR equation. Remnant-C was estimated as total cholesterol minus LDL-C minus HDL-C. Non-HDL-C was calculated as total cholesterol minus HDL-C.

### Determination of AIP and grouping

The calculation of AIP is determined by the base 10 logarithms of the ratio of the TG level to HDL-C level in molar concentration (mmol/L), and it is mathematically derived from log (TG/HDL-C) [14]. Subsequently, all patients were divided into MACE and non-MACE groups as well as the quartile groups based on their AIP values (Q1: < -0.064; Q2: -0.064–0.130; Q3: 0.130–0.328; Q4: > 0.328).

### Follow-up and clinical endpoints

In this study, all patients were followed up for a median duration of 35 months. Two trained physicians at Shanghai Tenth People’s Hospital recorded the clinical outcomes via telephone calls, outpatient visits, review of medical case history, and communication with patients’ families. The primary clinical endpoints of the present study were MACE, which is a combination of cardiovascular death, Ischemia-driven revascularization, nonfatal MI, heart failure, and nonfatal stroke. Deaths derived from heart failure, malignant arrhythmias, acute MI, or other cardiac conditions refer to cardiovascular death. Ischemia-driven revascularization was defined as revascularization due to continual angina or a positive test for cardiac ischemia. Nonfatal MI was defined as a composite of notable symptoms of myocardial ischemia, positive cardiac biomarkers, and observable dynamic changes on electrocardiograms [15]. The diagnosis of heart failure adhered to the latest guidelines provided by the ESC for the diagnosis and management of both acute and chronic heart failure [16]. The diagnosis of stroke is established based on the presence of cerebral infarction, as ascertained by the manifestation of characteristic clinical symptoms or through imaging examinations [17].

### Statistical analysis

We performed statistical analysis with the use of Statistical Package for Social Sciences (SPSS) v.25., and the figures were generated by GraphPad software 9. Ink. For continuous variables, the variables are displayed as the mean ± standard deviation, while the categorical variables were presented as counts and percentages (%). A t-test or an ANOVA was conducted to compare the continuous variables between groups. Pearson’s chi-squared (χ2) test or Fisher’s exact test, depending on the circumstance, was used to compare categorical variables.

Univariate Cox proportional hazards regression modeling was used to analyze independent clinical risk factors associated with MACE, and the clinical risk factors listed in Table 1 that probably facilitate the risk of adverse outcomes in CCS patients served as the variables in the univariate analysis stratified by the AIP quartile. All the significant covariates with P < 0.10 in the univariate analysis were further selected for the multivariate analysis to determine whether the AIP quartile can be served as the independent predictors for the MACE of the CCS patients, and the estimated hazard ratio (HR) and 95% confidence interval (CI) were applied in the analysis. The Kaplan–Meier method was used for the graphical evaluation of time-related MACE and differences were determined by log-rank tests. The Spearman correlation test was used to seek linear relations between AIP and other clinical risk factors. In addition, the receiver operating curve (ROC) analysis was utilized to calculate their corresponding area under the curve (AUC) and the optimal cut-off value of AIP to predict clinical outcomes according to the Youden index. All analysis was conducted two-sided, and a P-value < 0.05 was considered statistically significant for all analyses.

**Table 1:** Clinical characteristics of the study population stratified by MACE

	Total patients (N = 404)	MACE (N = 88)	non-MACE(N = 316)	P value
General characteristics				
Age (years)	63.61 ± 9.64	65.52 ± 9.22	63.08 ± 9.70	**0.035**
BMI (kg/m2)	25.05 ± 3.14	25.47 ± 3.04	24.94 ± 3.17	0.166
h (beats per minute)	76.70 ± 11.52	77.91 ± 10.79	76.36 ± 11.71	0.266
Male, n (%)	238(58.9)	55(62.5)	183(57.9)	0.439
SBP (mmHg)	134.52 ± 57.29	133.71 ± 20.09	134.73 ± 63.55	0.888
DBP (mmHg)	78.36 ± 12.79	77.05 ± 11.57	78.70 ± 13.08	0.304
Comorbidities				
DM, n (%)	144(35.6)	39(44.3)	105(33.2)	0.055
Hyperlipidemia, n (%)	127(31.4)	33(37.5)	94(29.7)	0.166
Atrial fibrillation, n (%)	20(4.9)	6(6.8)	14(4.4)	0.525
Smoke, n (%)	83(20.5)	20(22.7)	63(19.9)	0.567
Hypertension, n (%)	254(62.8)	56(63.6)	198(62.7)	0.867
Heart failure, n (%)	5(1.2)	3(3.4)	2(0.6)	0.120
CKD, n (%)	30(7.4)	9(10.2)	21(6.6)	0.257
Stroke, n (%)	62(15.3)	19(21.6)	43(13.6)	0.066
PCI conducted, n (%)	198(49.0)	43(48.9)	155(49.1)	0.622
1-vessel disease	110(27.2)	24(27.3)	86(27.2)	0.991
2-vessel disease	90(22.3)	21(23.9)	69(21.8)	0.686
3-vessel disease	57(14.1)	16(18.2)	41(13.0)	0.215
Laboratory parameters				
TC (mmol/L)	3.83 ± 1.02	3.93 ± 1.06	3.80 ± 1.01	0.295
HDL-C (mmol/L)	1.09 ± 0.27	1.05 ± 0.27	1.10 ± 0.27	0.137
LDL-C (mmol/L)	2.10 ± 0.90	2.17 ± 0.95	2.08 ± 0.89	0.430
TG (mmol/L)	1.76 ± 1.37	1.92 ± 1.46	1.71 ± 1.35	0.208
Remnant-C (mmol/L)	0.64 ± 0.50	0.71 ± 0.43	0.62 ± 0.52	0.129
Non-HDL (mmol/L)	2.73 ± 0.99	2.88 ± 1.00	2.69 ± 0.99	0.112
AIP	0.15 ± 0.29	0.20 ± 0.28	0.13 ± 0.30	**0.039**
FBG (mmol/L)	5.75 ± 1.76	6.23 ± 2.33	5.62 ± 1.55	**0.025**
Hb1AC (%)	6.36 ± 1.20	6.53 ± 1.14	6.31 ± 1.21	0.147
CRP (mg/L)	3.94 ± 6.94	4.71 ± 7.14	3.71 ± 6.88	0.247
SCr (umol/L)	75.21 ± 20.80	78.45 ± 17.34	74.29 ± 21.62	0.097
ALT (U/L)	24.22 ± 17.87	27.12 ± 27.61	23.41 ± 13.94	0.228
AST (U/L)	23.56 ± 18.34	28.60 ± 34.86	22.13 ± 9.03	0.088
eGFR (ml/min/l.73 m^2^)	103.94 ± 27.35	96.82 ± 23.69	105.95 ± 28.01	**0.006**
Hb (g/L)	134.02 ± 15.59	134.63 ± 14.56	133.85 ± 15.89	0.682
Platelets(x10^9^/L)	205.37 ± 52.62	208.88 ± 54.42	204.39 ± 52.16	0.480
LVEF (%)	61.88 ± 6.06	59.06 ± 9.47	62.72 ± 4.29	**0.001**
Cardiovascular medical therapy				
Aspirin, n (%)	290(71.8)	56(63.6)	234(74.1)	0.055
Clopidogrel, n (%)	228(56.4)	57(64.8)	171(54.1)	0.075
Statin, n (%)	360(89.1)	79(89.8)	281(88.9)	0.821
ACEI/ARB, n (%)	183(45.3)	49(55.7)	134(42.4)	**0.027**
Beta blocker, n (%)	194(48.0)	39(44.3)	155(49.1)	0.432
CCB, n (%)	160(39.6)	39(44.3)	121(38.3)	0.307

*MACE* major adverse cardiovascular event, *BMI* body mass index, *HR* heart rate, *SBP* systolic blood pressure, *DBP* diastolic blood pressure, *DM* diabetes mellitus, *CKD* chronic kidney disease, *PCI* percutaneous coronary intervention, *TC* total cholesterol, *HDL-C* high-density lipoprotein-cholesterol, *LDL-C* low-density lipoprotein-cholesterol, *TG* triglyceride, *AIP* atherogenic index of plasma, *FBG* fasting blood glucose, *HbA1c* hemoglobin A1c, *CRP* C-reactive protein, *SCr* serum creatine, *ALT* alanine aminotransferase, *AST* aspartate aminotransferase, *eGFR* estimated glomerular filtration rate, *Hb* hemoglobin, *LVEF* left ventricular ejection fraction, *ACEI/ARB* angiotensin-converting-enzyme inhibitor/angiotensin receptor blocker, *CCB* calcium channel blocker

## Results

### Baseline characteristics

**Fig. 1:**
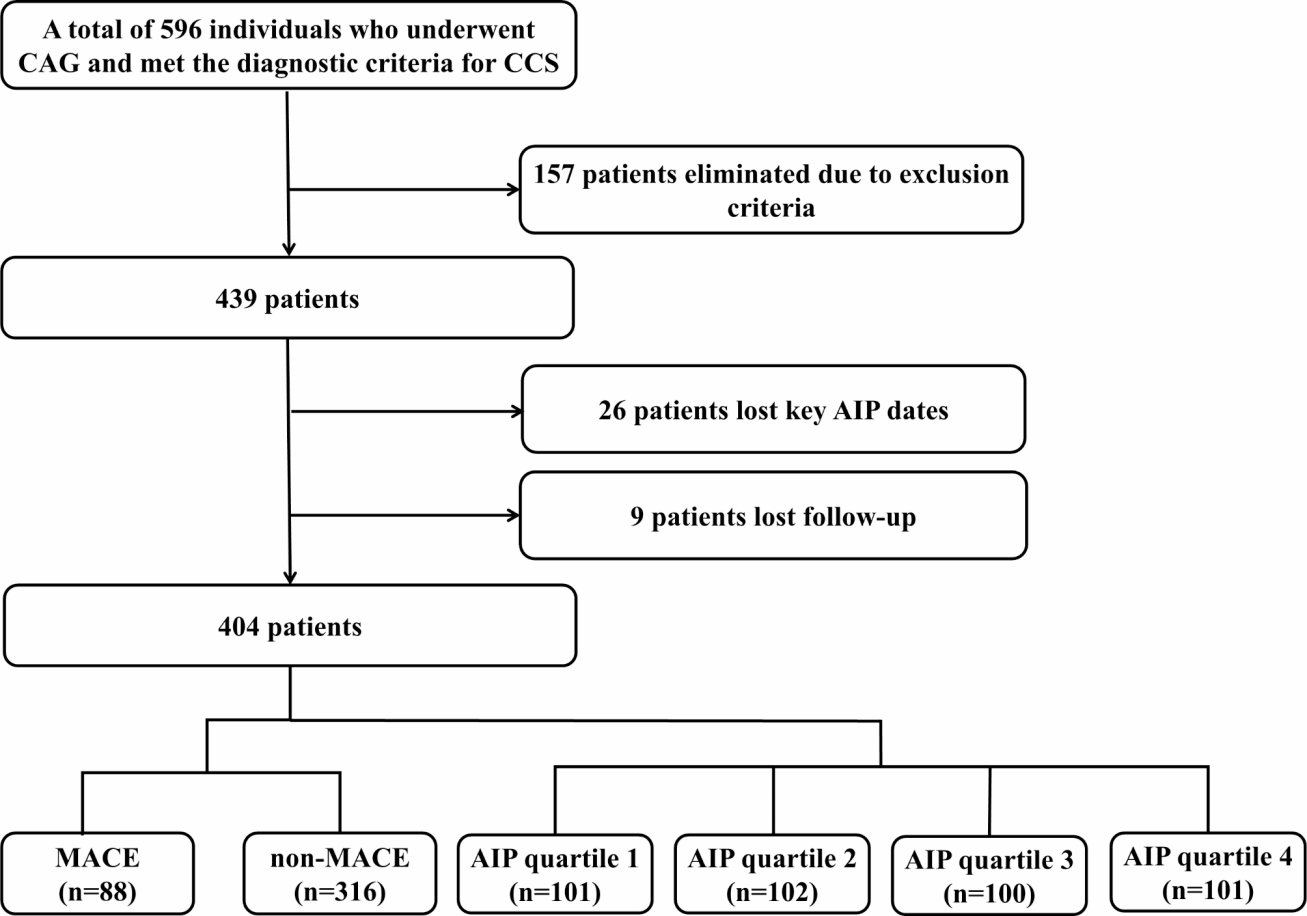
Flow diagram of study population ***CAG*** coronary angiography, ***CCS*** chronic coronary syndrome, ***AIP*** atherogenic index of plasma, ***MACE*** major adverse cardiovascular event

A total of 596 individuals who underwent CAG and met the diagnostic criteria for CCS were included in the study, with 157 patients eliminated due to exclusion criteria, 9 patients lost to follow-up, and 26 patients missing key AIP dates. Finally, 404 individuals were included in the final analysis of the present study (Fig. 1). Among these, 144 (35.6%) patients had diabetes mellitus (DM).

In Table 1, among all patients, 88 patients developed MACE after a median follow-up of 35 months. Between patients who developed MACE and patients who did not, there were significant differences in AIP value (0.20 ± 0.28 vs. 0.13 ± 0.30; P = 0.039). In addition, we observed that the age and FBG levels were also significantly higher in the MACE group, while the LVEF, eGFR, and ACEI/ARB use were significantly lower in the MACE group. Except for these, no differences were found between the MACE and non-MACE groups regarding other baseline characteristics or laboratory findings (all P > 0.05).

Subsequently, the patients were divided into 4 groups based on the AIP quartiles (Table 2). We discovered that the patients with higher AIP values (Q4 group) tended to have higher BMI, diabetes and hyperlipidemia. The blood parameters including TG, TC, remnant-C, non-HDL, Hb1AC, Hb, ALT, and FBG in the Q4 group were significantly higher than those in the Q1, Q2, and Q3 groups, whereas the HDL-C was lower with the increasing of AIP values.

**Table 2:** Clinical characteristics of the study population stratified by AIP quartile

	Total patients (N = 404)	Q1 (N = 101)	Q2 (N = 102)	Q3 (N = 100)	Q4 (N = 101)	P value
General characteristics						
Age (years)	63.61 ± 9.64	66.02 ± 10.29	64.16 ± 9.19	61.67 ± 8.89	62.57 ± 9.70	**0.008**
BMI (kg/m2)	25.05 ± 3.14	23.72 ± 2.98	25.14 ± 3.13	25.37 ± 3.00	25.98 ± 3.05	**< 0.001**
h (beats per minute)	76.70 ± 11.52	76.80 ± 12.25	76.10 ± 11.88	76.05 ± 9.02	77.85 ± 12.61	0.658
Male, n (%)	238(58.9)	51(50.5)	58(56.9)	63(63.0)	66(65.3)	0.136
SBP (mmHg)	134.52 ± 57.29	140.51 ± 110.32	132.54 ± 20.51	132.14 ± 17.58	133.04 ± 21.71	0.710
DBP (mmHg)	78.36 ± 12.79	75.96 ± 12.59	78.41 ± 12.04	79.64 ± 13.09	79.39 ± 13.29	0.175
Comorbidities						
DM, n (%)	144(35.6)	25(24.8)	38(37.3)	37(37.0)	44(43.6)	**0.042**
Hyperlipidemia, n (%)	127(31.4)	10(9.9)	13(12.7)	26(26.0)	78(77.2)	**< 0.001**
Atrial fibrillation, n (%)	20(5.0)	4(4.0)	4(3.9)	2(2.0)	10(9.9)	0.082
Smoke, n (%)	83(20.5)	13(12.9)	23(22.5)	22(22.0)	25(24.8)	0.163
Hypertension, n (%)	254(62.9)	57(56.4)	68(66.7)	62(62.0)	67(66.3)	0.396
Heart failure, n (%)	5(1.2)	2(2.0)	1(1.0)	0(0.0)	2(2.0)	0.701
CKD, n (%)	30(7.4)	8(7.9)	4(3.9)	9(9.0)	9(8.9)	0.435
Stroke, n (%)	62(15.3)	16(15.8)	11(10.8)	16(16.0)	19(18.8)	0.454
PCI, n (%)	198(51.3)	50(52.6)	53(53.5)	45(45.0)	50(53.2)	0.674
1-vessel disease	110(27.2)	29(28.7)	27(26.5)	26(26.0)	28(27.7)	0.973
2-vessel disease	90(22.3)	20(19.8)	27(26.5)	17(17.0)	26(25.7)	0.295
3-vessel disease	57(14.1)	18(17.8)	12(11.8)	14(14.0)	13(12.9)	0.628
Laboratory parameters						
TC (mmol/L)	3.83 ± 1.02	3.65 ± 0.89	3.67 ± 1.01	3.98 ± 1.12	4.02 ± 1.01	**0.010**
HDL-C (mmol/L)	1.09 ± 0.27	1.37 ± 0.24	1.11 ± 0.21	1.01 ± 0.18	0.87 ± 0.17	**< 0.001**
LDL-C (mmol/L)	2.10 ± 0.90	1.90 ± 0.77	2.04 ± 0.90	2.32 ± 1.02	2.15 ± 0.85	**0.008**
TG (mmol/L)	1.76 ± 1.37	0.86 ± 0.20	1.22 ± 0.25	1.72 ± 0.38	3.22 ± 2.02	**< 0.001**
Remnant-C (mmol/L)	0.64 ± 0.50	0.38 ± 0.24	0.55 ± 0.31	0.65 ± 0.28	0.99 ± 0.75	**< 0.001**
Non-HDL (mmol/L)	2.73 ± 0.99	2.28 ± 0.80	2.56 ± 0.89	2.97 ± 1.03	3.11 ± 1.02	**< 0.001**
AIP	0.15 ± 0.29	-0.21 ± 0.11	0.04 ± 0.05	0.23 ± 0.06	0.53 ± 0.20	**< 0.001**
FBG (mmol/L)	5.75 ± 1.76	5.40 ± 1.01	5.48 ± 1.24	5.70 ± 1.44	6.44 ± 2.69	**< 0.001**
Hb1AC (%)	6.36 ± 1.20	6.03 ± 0.61	6.28 ± 0.99	6.33 ± 1.18	6.82 ± 1.65	**< 0.001**
CRP (mg/L)	3.94 ± 6.94	4.19 ± 6.80	3.72 ± 5.71	4.05 ± 9.36	3.79 ± 5.25	0.962
SCr (umol/L)	75.21 ± 20.80	73.09 ± 17.85	73.36 ± 21.17	78.12 ± 25.11	76.35 ± 18.19	0.255
ALT (U/L)	24.22 ± 17.87	19.66 ± 14.46	26.71 ± 23.83	24.89 ± 14.11	25.60 ± 16.47	**0.026**
AST (U/L)	23.56 ± 18.34	22.82 ± 24.62	25.02 ± 22.27	22.49 ± 9.49	23.85 ± 12.23	0.764
eGFR (ml/min/l.73 m)	103.94 ± 27.35	105.85 ± 27.44	106.62 ± 26.46	100.95 ± 27.46	102.19 ± 28.03	0.386
Hb (g/L)	134.02 ± 15.59	131.10 ± 12.65	131.41 ± 13.72	135.51 ± 19.43	138.07 ± 14.85	**0.003**
Platelets(x10^9^/L)	205.37 ± 52.64	203.39 ± 50.47	202.70 ± 52.27	204.01 ± 52.53	211.36 ± 55.43	0.649
LVEF (%)	61.88 ± 6.06	61.42 ± 8.09	62.03 ± 5.14	62.58 ± 5.42	61.53 ± 5.01	0.506
Cardiovascular medical therapy						
Aspirin, n (%)	290(71.8)	66(65.3)	70(68.6)	76(76.0)	78(77.2)	0.177
Clopidogrel, n (%)	228(56.4)	62(61.4)	60(58.8)	49(49.0)	57(56.4)	0.322
Statin, n (%)	360(89.1)	91(90.1)	93(91.2)	86(86.0)	90(89.1)	0.671
ACEI/ARB, n (%)	183(45.3)	48(47.5)	48(47.1)	41(41.0)	46(45.5)	0.782
Beta blocker, n (%)	194(48.0)	48(47.5)	53(52.0)	44(44.0)	49(48.5)	0.729
CCB, n (%)	160(39.6)	38(37.6)	43(42.2)	40(40.0)	39(38.6)	0.921

*AIP* atherogenic index of plasma, *Q1* AIP quartile 1, *Q2* AIP quartile 2, *Q3* AIP quartile 3, *Q4* AIP quartile 4, *BMI* body mass index, *HR* heart rate, *SBP* systolic blood pressure, *DBP* diastolic blood pressure, *DM* diabetes mellitus, *CKD* chronic kidney disease, *PCI* percutaneous coronary intervention, *TC* total cholesterol, *HDL-C* high-density lipoprotein-cholesterol, *LDL-C* low-density lipoprotein-cholesterol, *TG* triglyceride, *FBG* fasting blood glucose, *HbA1c* hemoglobin A1c, *CRP* C-reactive protein, *SCr* serum creatine, *ALT* alanine aminotransferase, *AST* aspartate aminotransferase, *eGFR* estimated glomerular filtration rate, *Hb* hemoglobin, *LVEF* left ventricular ejection fraction, *ACEI/ARB* angiotensin-converting-enzyme inhibitor/angiotensin receptor blocker, *CCB* calcium channel blocker

The information of lipid parameters and antidiabetic drugs of patients with DM are presented in Additional file 1: Table S1.

### Association between clinical outcomes and AIP quartile

After a median duration of 35 months follow-up, a total of 88 patients (21.8%) developed MACE, which include 7 (1.7%) cardiovascular death, 29 (7.2%) Ischemia-driven revascularization, 5 (1.2%) nonfatal MI, 27 (6.7%) heart failure and 20 (5.0%) nonfatal strokes. In comparison to patients displaying lower AIP values (Q1, Q2, and Q3 groups), those belonging to the Q4 group of patients tended to have a higher rate of MACE (31.7% vs. 16.8%, 15.7%, and 23.0% respectively; P = 0.023), as illustrated in Table 3.

**Table 3:** Clinical outcomes of study population according to AIP quartile

	AIP quartile				P-value
	Q1(N = 101)	Q2(N = 102)	Q3(N = 100)	Q4(N = 101)	
MACE	17(16.8)	16(15.7)	23(23.0)	32(31.7)	**0.023**
Cardiovascular death	1(1.0)	1(1.0)	2(2.0)	3(3.0)	0.664
Ischemia-driven revascularization	4(4.0)	6(5.9)	9(9.0)	10(9.9)	0.328
Nonfatal MI	1(1.0)	1(1.0)	0(0.0)	3(3.0)	0.222
Heart failure	8(7.9)	4(3.9)	8(8.0)	7(6.9)	0.619
Nonfatal stroke	3(3.0)	4(3.9)	4(4.0)	9(8.9)	0.243

*AIP* atherogenic index of plasma, *Q1* AIP quartile 1, *Q2* AIP quartile 2, *Q3* AIP quartile 3, *Q4* AIP quartile 4, *MACE* major adverse cardiovascular event, *MI* myocardial infarction

**Fig. 2:**
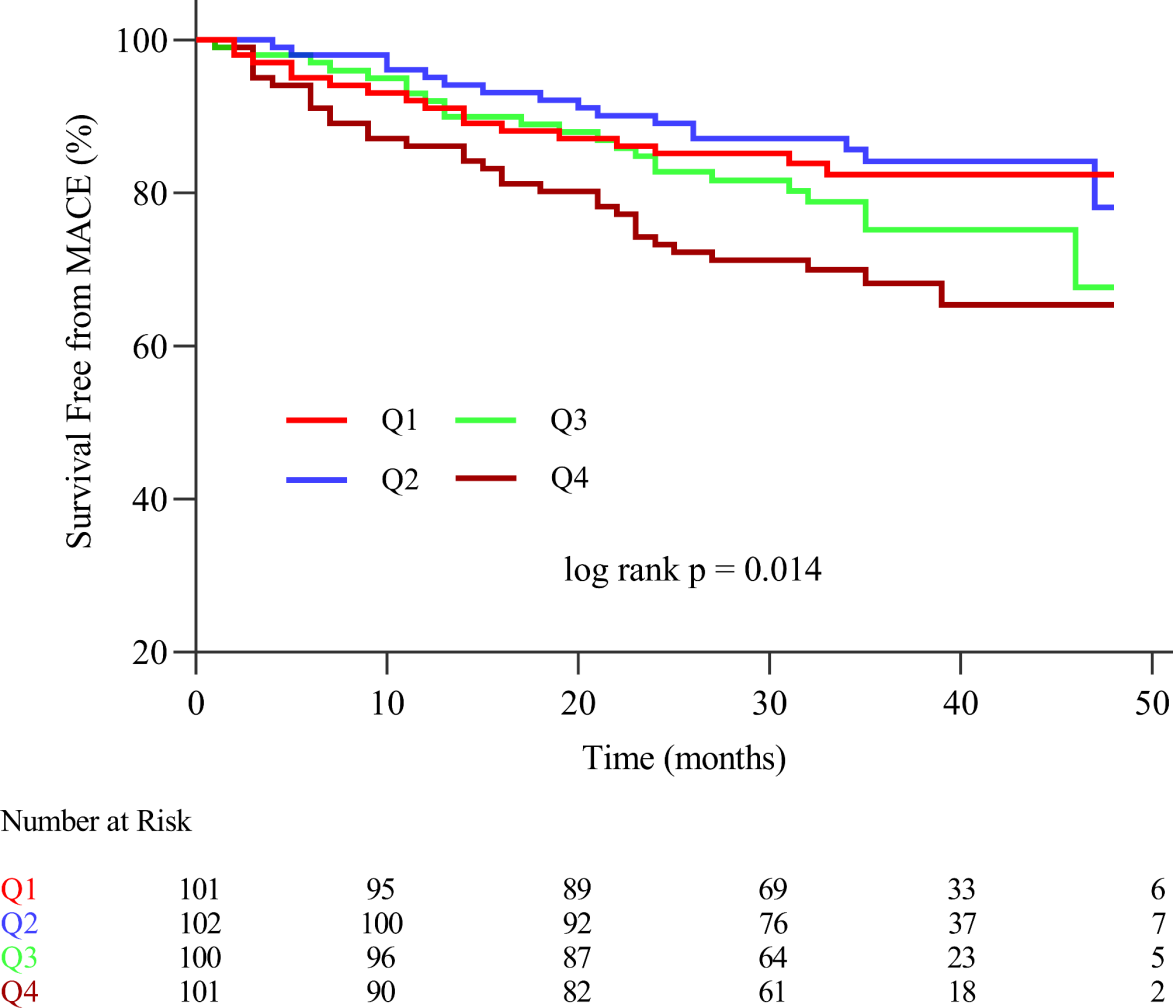
Kaplan–Meier survival curve for MACE in patients with CCS according to AIP quartile ***MACE*** major adverse cardiovascular events, ***Q1*** AIP quartile 1, ***Q2*** AIP quartile 2, ***Q3*** AIP quartile 3, ***Q4*** AIP quartile 4

To perform overall survival analysis, the log-rank test was employed to compare the Kaplan-Meier curve among the targeted patients, as depicted in Fig. 2. The analysis demonstrated that patients in the Q4 group exhibited the highest MACE risk when compared to the remaining patients in the other groups (log-rank P = 0.014).

The results of the Kaplan-Meier curve for each component of MACE (cardiovascular death, ischemia-driven revascularization, nonfatal MI, heart failure, and nonfatal stroke) are shown in Additional file 1: Figure S1.

### Predictive factors of MACE

The findings from both univariate and multivariate Cox regression analyses of MACE are presented in Table 4. In the univariate Cox regression analysis, it was observed that patients in the Q4 group exhibited a 2.081-fold increased risk of MACE in comparison to those in the Q1 group (HR, 2.081; 95% CI 1.155–3.752; P = 0.015). Furthermore, within the univariate regression analysis, FBG, LVEF, history of DM, and heart failure were identified as predictive factors for MACE.

**Table 4:** Cox regression analysis for MACE of study population

	Univariate analysis HR (95% CI)	P value	Multivariate analysis HR (95% CI)	P value
AIP	2.088(1.066–4.088)	**0.032**	0.213(0.031–1.485)	0.119
age	1.022(1.000-1.046)	0.052	1.029(1.002–1.056)	**0.032**
BMI	1.049(0.983–1.119)	0.147		
Heart rate	1.009(0.992–1.026)	0.305		
Male	1.259(0.818–1.939)	0.296		
TC	1.117(0.917–1.361)	0.271		
HDL-C	0.553(0.237-1.200)	0.129		
LDL-C	1.098(0.876–1.377)	0.416		
TG	1.085(0.965–1.221)	0.172		
Remnant-C	1.378(0.938–2.026)	0.102		
Non-HDL	1.185(0.968–1.451)	0.101		
AIP quartile				
Q1	Reference			
Q2	0.889(0.449–1.759)	0.735	1.463(0.622–3.444)	0.383
Q3	1.409(0.752–2.637)	0.284	3.703(1.287–10.660)	**0.015**
Q4	2.081(1.155–3.752)	**0.015**	7.892(1.818–34.269)	**0.006**
FBG	1.126(1.035–1.225)	**0.006**	1.062(0.949–1.189)	0.292
Hb1AC	1.113(0.961–1.289)	0.153		
CRP	1.012(0.991–1.033)	0.255		
Hb	1.004(0.990–1.018)	0.571		
Platelets	1.001(0.997–1.005)	0.479		
LVEF	0.916(0.893–0.940)	**< 0.001**	0.910(0.883–0.938)	**< 0.001**
DM	1.538(1.010–2.343)	**0.045**	1.279(0.768–2.131)	0.344
Hyperlipidemia	1.439(0.933–2.218)	0.099	0.909(0.488–1.693)	0.764
Atrial fibrillation	1.450(0.633–3.322)	0.380		
Smoke	1.154(0.701-1.900)	0.573		
Hypertension	1.035(0.670–1.598)	0.877		
Heart failure	4.411(1.392–13.982)	**0.012**	0.697(0.188–2.581)	0.589
CKD	1.510(0.758–3.010)	0.241		
Stroke	1.479(0.890–2.459)	0.131		
PCI	1.148(0.739–1.781)	0.539		
1-vessel disease	0.974(0.609–1.558)	0.914		
2-vessel disease	1.107(0.678–1.808)	0.683		
3-vessel disease	1.461(0.850–2.513)	0.170		
Statins	1.064(0.534–2.121)	0.860		

* h* hazard ratio, *CI* confidence interval, *MACE* major adverse cardiovascular event, *BMI* body mass index, *HR* heart rate, *SBP* systolic blood pressure, *DBP* diastolic blood pressure, *TC* total cholesterol, *HDL-C* high-density lipoprotein-cholesterol, *LDL-C* low-density lipoprotein-cholesterol, *TG* triglyceride, *Q1* AIP quartile 1, *Q2* AIP quartile 2, *Q3* AIP quartile 3, *Q4* AIP quartile 4, *AIP* atherogenic index of plasma, *FBG* fasting blood glucose, *HbA1c* hemoglobin A1c, *CRP* C-reactive protein, *SCr* serum creatine, *ALT* alanine aminotransferase, *AST* aspartate aminotransferase, *eGFR* estimated glomerular filtration rate, *Hb* hemoglobin, *LVEF* left ventricular ejection fraction, *DM* diabetes mellitus, *CKD* chronic kidney disease, *PCI* percutaneous coronary intervention, *CCB* Calcium Channel Blockers

Subsequently, upon adjusting for potential confounders such as age, FBG, LVEF, diabetes, hyperlipidemia, and heart failure, multivariate Cox regression analyses showed that the incidence of MACE in the Q4 group remained significantly elevated (adjusted HR,7.892; 95% CI1.818-34.269; P = 0.006).

Ulteriorly, subgroup analysis was performed to evaluate possible impact of relevant paraments on associations between AIP and MACE risk (Additional file 1: Table S2). The results showed a stronger association between AIP and MACE risk in younger, male, hypertensive, hsCRP < 10, and non-diabetes CCS patients. Besides, the analysis of antidiabetic drugs in DM patients with CCS in Additional file 1: Table S3 revealed insulin, metformin, glinides, sulfonylureas, and alpha-glucosidase inhibitors had no impact on the association of AIP with MACE risk either.

### Correlation between the AIP and other risk factors

To explore potential relationships between AIP and other clinical risk factors, a Spearman correlation test was conducted. The results indicated significant correlations between AIP and several variables, including age (r =-0.174; p < 0.001), BMI (r = 0.275; p < 0.001), diastolic blood pressure (DBP) (r = 0.124; p = 0.015), FBG (r = 0.157; p = 0.002), Hb1AC (r = 0.196; p < 0.001), SCr (r = 0.133; p = 0.008), TC (r = 0.166; p = 0.001), HDL-C (r =-0.697; p < 0.001), LDL-C (r = 0.124; p = 0.013), and TG (r = 0.932; p < 0.001), as detailed in Table 5. Importantly, AIP is also correlated well with age (r =-0.193; p = 0.021), FBG (r = 0.206; p = 0.015), TC (r = 0.288; p < 0.001), HDL-C (r =-0.640; p < 0.001), LDL-C (r = 0.220; p = 0.009), and TG (r = 0.948; p < 0.001) in CCS patients with DM (Additional file 1: Table S4).

**Table 5:** Correlation between the atherogenic index of plasma and other variables

Variable	Coefficient	P value
Age	-0.174	**< 0.001**
BMI	0.275	**< 0.001**
SBP	0.061	0.235
DBP	0.124	**0.015**
FBG	0.157	**0.002**
Hb1AC	0.196	**< 0.001**
SCr	0.133	**0.008**
TC	0.166	**0.001**
HDL-C	-0.697	**< 0.001**
LDL-C	0.124	**0.013**
TG	0.932	**< 0.001**
LVEF	-0.064	0.211

*BMI* body mass index, *SBP* systolic blood pressure, *DBP* diastolic blood pressure, *FBG* fasting blood glucose, *HbA1c* hemoglobin A1c, *SCr* serum creatine, *TC* total cholesterol, *HDL-C* high-density lipoprotein-cholesterol, *LDL-C* low-density lipoprotein-cholesterol, *TG* triglyceride, *LVEF* left ventricular ejection fraction

### The optimal cut-off value of AIP for predicting outcomes among CCS

**Fig. 3:**
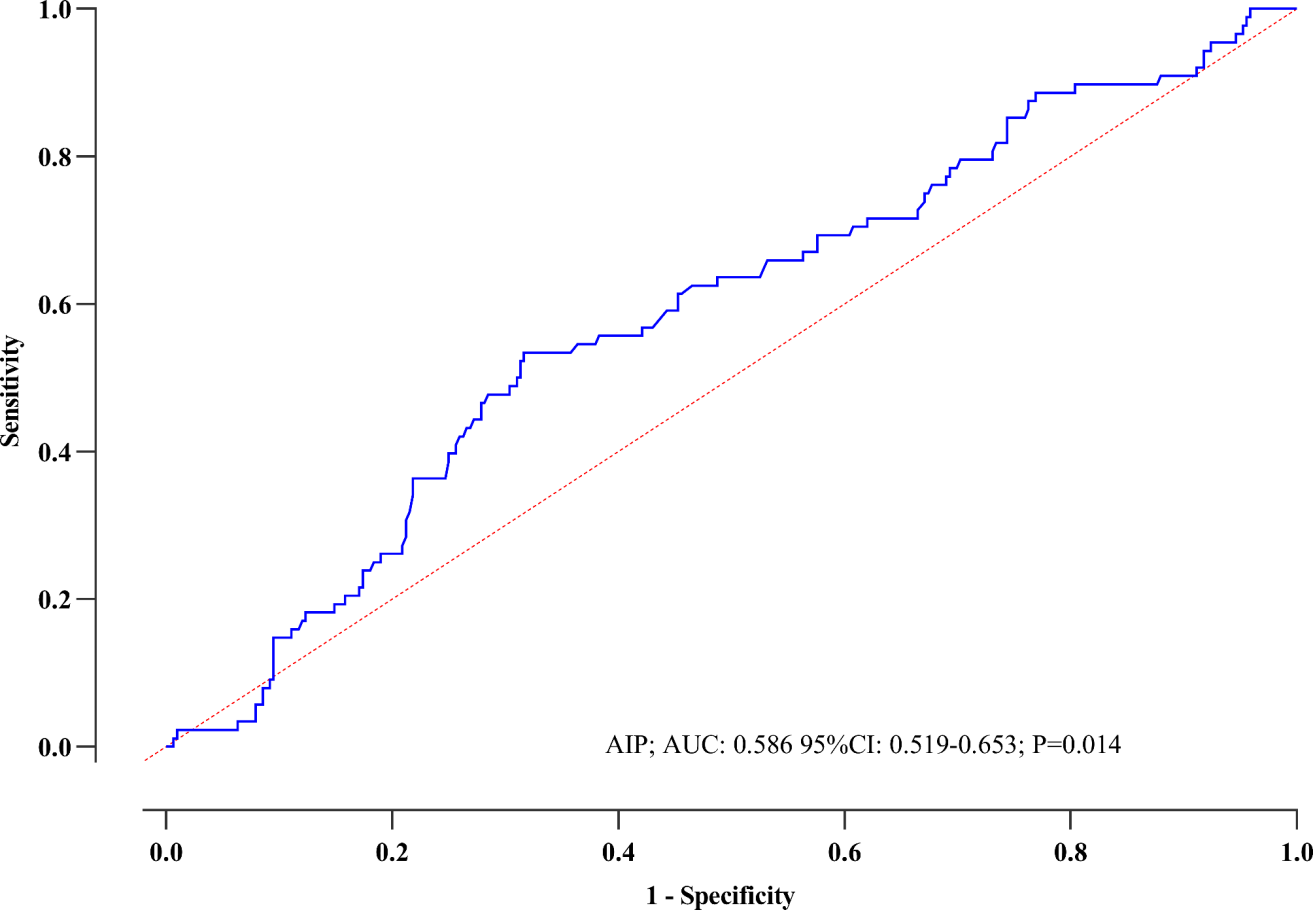
Receiver operating characteristic analysis of the ability of the AIP to predict MACE in study population ***AUC*** area under the curve, ***AIP*** atherogenic index of plasma, ***CI*** confidence interval

Figure 3 illustrates the ROC curve analysis of the AIP for prognosticating MACE in patients diagnosed with CCS. The optimal cut-off value of AIP identified for predicting CCS was 0.24, yielding an AUC of 0.586 (95% CI: 0.519–0.653; P = 0.014). This analysis substantiates the favorable predictive accuracy of AIP concerning prognosis.

## Discussion

The present study is the first to examine the correlation between AIP and the prognostic implications for patients with CCS. The novel discoveries from our investigation encompassed the following observations: (1) a significant elevation in AIP levels among patients who experienced MACE in contrast to those who did not encounter such events; (2) elevated AIP level was found to be independently associated with an increased risk of MACE in patients diagnosed with CCS; (3) the AIP emerged as a significant independent risk predictor for CCS patients, with a discernible optimal cut-off value of 0.24 for predicting the occurrence of MACE.

CCS represents a clinical manifestation of CAD characterized by the exclusion of acute coronary thrombosis [4]. Despite significant developments in the prevention and treatment of atherosclerosis, CAD remains the leading cause of death among the Chinese and global populations, and its incidence is rising and starting earlier in life [18–21]. Given the unfavorable prognosis often associated with the CCS population, the utilization of clinical risk predictors becomes pivotal in identifying CCS patients at heightened risk for new major cardiovascular clinical outcomes. Liu et al. reported that N-terminal pro-brain natriuretic peptide (NT-proBNP) could well predict worse outcomes in dysglycemic patients with CCS and normal left-ventricular systolic function, suggesting that NT-proBNP may help with risk stratification in this population [22]. Guo et al. suggested that the triglyceride-glucose (TyG) index could be a potent predictor in evaluating the prognosis of CCS patients undergoing PCI, and has shown that increased TyG index was associated with elevated risk for long-term PCI complications, including repeat revascularization and in-stent restenosis [23]. Our recent study demonstrated TyG index emerged as an independent predictor of MACE among patients with CCS and coronary microvascular dysfunction (CMD) either [24]. In addition, our recent study highlighted that CMD evaluated through the CAG-derived index of microvascular resistance emerged as a significant and independent predictor of MACE among patients with diabetes diagnosed with CCS [25]. Notwithstanding, there remains a paucity of research examining the predictive capability of a novel index within the CCS population. Our study identifies a novel predictor of MACE in patients diagnosed with CCS, with the ultimate aim of implementing preventive strategies to impede disease progression.

Elevated triglyceride levels independently raise the risk of atherosclerosis and CCS. Impaired triglyceride metabolism can lead to the formation of atherogenic triglyceride-rich lipoproteins (TRLs) and small, dense LDL particles, promoting atherosclerosis. Impaired triglyceride metabolism is a component of metabolic syndrome, which increases CCS risk. In addition, it is known that higher HDL-C levels are associated with protective effects against atherosclerosis, while higher triglyceride levels are linked to increased cardiovascular risk. Lower HDL-C levels may inhibit the anti-atherogenic properties of HDL, and these lipid profile changes may precede the development of glycemic dysregulation [26–28]. AIP, represented by the logarithm of the ratio of plasma concentrations of TG to HDL-C, is a pivotal index that holds promise as an independent parameter for estimating cardiac risk [9, 29]. Lipids and their lipoprotein constituents have been identified as both mediators and markers of CAD, denoted by an elevated ratio of LDL-C to HDL-C and an increased level of TG [30]. Numerous clinical studies have consistently revealed that the AIP exhibits robust predictive potential for adverse outcomes among patients with CVD, thereby positioning it as a noteworthy indicator for atherosclerosis prediction [11, 31]. Süleymanoğlu et al. revealed that AIP was an independent predictor for no-reflow in patients with acute STEMI who underwent PCI [12]. Furthermore, multiple investigations have also indicated that the AIP serves as a significant risk factor for CAD in patients diagnosed with type 2 diabetes mellitus (T2DM) [32, 33]. The study conducted by Wan et al. unveiled that heightened AIP values emerge as a robust and independent prognostic indicator for all-cause mortality and subsequent CVD following coronary revascularization [34]. Nonetheless, the prognostic significance of AIP within the CCS population remains obscure, rendering the identification of high-risk patients of paramount clinical importance. In accordance with prior investigations, the present study elucidates a strong association between AIP and the risk of MACE in patients diagnosed with CCS. The clinical outcomes of CCS patients exhibiting elevated levels of AIP demonstrated a higher incidence of MACE even following adjustment for other potential confounding risk factors. This underscores the significance of lipid distribution, as evidenced by the AIP index, in the pathophysiological mechanisms underlying CCS [9]. Significantly, in our investigation, we observed a substantial correlation between the AIP and a range of pertinent variables, including Age, BMI, DBP, FBG, Hb1AC, SCr, TC, HDL-C, LDL-C, and TG, all of which have been previously identified in as relevant risk indicators for CVD [35–42]. These findings underscore the significance of AIP as a determinant of disease severity and its substantial prognostic impact in individuals with CCS.

The determination of a predictive cut-off value for AIP varies across different diseases owing to the diversity in risk factors and pathophysiological mechanisms associated with each specific condition. Prior investigations have conducted multiple studies focusing on the sensitivity and specificity of diverse cut-off values for AIP in patients with CAD [12, 14, 43–46]. Khosravi et al. Reported that utilizing the AIP index alone can serve as an effective predictor of atherogenic plaque instability and the best cut-off value of AIP was 0.62, with a sensitivity of 89.70% and specificity of 34% [43]. As reported in a previous study, employing a cut-off level of 0.54, the AIP exhibited a sensitivity of 46.02% and a specificity of 84.73% in detecting the no-reflow phenomenon in patients with acute STEMI who underwent PCI [12]. An additional investigation demonstrated the favorable predictive accuracy of AIP in forecasting post-PCI outcomes in patients with T2DM. Consequently, monitoring AIP levels for lipid management in diabetic patients after PCI is recommended, with the target threshold set at 0.318, as the baseline AIP value of 0.318 was identified as the optimal cut-off point for prognostic risk prediction [14]. In the investigation by Karadağ et al., it was observed that AIP serves as a predictor of ejection fraction and possesses specific cut-off values for effectively diagnosing heart failure (HF); notably, the identified cut-off level of 0.47 exhibited a sensitivity of 68% and a specificity of 53% in the context of HF diagnosis [44]. Among patients diagnosed with STEMI, the AIP emerged as a significant marker influencing pre-PCI thrombolysis in myocardial infarction flow; the established cut-off value was determined to be 0.59, with corresponding sensitivity and specificity rates of 67.6% and 68.4%, respectively [45]. In a recent study investigating prognostic risk factors for acute myocardial infarction (AMI), the optimal cut-off value for the AIP concerning AMI was identified as -0.06142 [46]. Nevertheless, research regarding the optimal cut-off value of AIP for predicting MACE among patients with CCS remains unknown. Our ROC curve analysis of AIP indicated that the most suitable cut-off value for predicting MACE within the CCS population was 0.24, with an AUC of 0.586. We further divided the patients into a high AIP group and a low AIP group based on our own cut-off value of 0.24; the results show that the MACE rate is higher in the high AIP group (AIP ≥ 0.24) compared to the low AIP group (AIP < 0.24) (Additional file 1: Figure S2). This finding suggests that the AIP exhibits favorable predictive accuracy concerning prognosis.

Overall, the findings of this study may contribute to the existing body of knowledge on the role of AIP in cardiovascular diseases and provide insights into the potential clinical utility of AIP as a marker for CCS. Ultimately, this study may have implications for improving risk assessment, prevention, and management strategies for patients with CCS, leading to better patient outcomes and reduced burden of the disease.

### Study limitations

Several limitations are associated with our study. Firstly, it is important to acknowledge that this research constitutes a single-center retrospective observational study, which inherently imposes certain restrictions on the generalizability of the findings. Secondly, the sample size employed in the study is relatively small, potentially limiting the statistical power and precision of the results. Additionally, the follow-up period of 35 months might be considered relatively short in the context of CCS, possibly affecting the completeness of long-term outcomes. Thirdly, in our study, the lipid parameters employed for calculating the AIP were assessed at the time of admission and not continuously monitored throughout the follow-up period. Moreover, it is essential to acknowledge that all participants in this study are of Chinese ethnicity; although racial homogeneity might be considered an advantage, the findings of this study may not be extrapolated to other ethnic groups without caution. Given the observational nature of the study, we cannot confidently establish definitive conclusions about causative mechanisms and temporal relationships. Despite these limitations, our findings indicate that patients with elevated AIP had an independent relationship with worse prognosis. An elevated AIP value indicates to medical practitioners that patients are more likely to be at a significant risk of metabolic dysfunction. This condition involves a critical concern regarding serum lipid management, necessitating immediate adjustments to their lifestyle. Further studies are required to highlight this association of AIP with MACE in a larger cohort with multi-center prospective studies and elucidate the precise mechanism of AIP in CCS.

## Conclusion

This study indicates, for the first time, that higher risk in CCS patients is strongly related to increased AIP. AIP is an independent predictor of MACE in CCS, and its optimal cut-off value is 0.24. These findings collectively highlight the imperative clinical significance of AIP in the realm of early risk prediction and preventive strategies concerning CCS. Further studies are required to elucidate its precise mechanism in CCS patients along with other triglyceride-rich lipoproteins and their remnants.

### Electronic supplementary material

Below is the link to the electronic supplementary material.

Supplementary Material 1

## Data Availability

The data analyzed in this study can be obtained from the corresponding author with a reasonable request.

## Abbreviations

AIP: atherogenic index of plasma
CCS: chronic coronary syndromes
MACE: major adverse cardiovascular events
CAD: coronary artery disease
ACS: acute coronary syndromes
TG: triglycerides
HDL-C: high-density lipoprotein-cholesterol
LDL-C: low-density lipoprotein-cholesterol
VLDL: very low-density lipoprotein
CVD: cardiovascular disease
STEMI: ST-segment elevation myocardial infarction
PCI: percutaneous coronary intervention
MI: myocardial infarction
CABG: coronary artery bypass graft surgery
LVEF: left ventricular ejection fraction
BMI: body mass index
CKD: chronic kidney disease
FBG: fasting blood glucose
HbA1c: hemoglobin A1c
TC: total cholesterol
CRP: C-reactive protein
ALT: alanine aminotransferase
AST: aspartate aminotransferase
eGFR: estimated glomerular filtration rate
Hb: hemoglobin
MDRD: modification of diet in renal disease study
HR: hazard ratio
DBP: diastolic blood pressure
NT-proBNP: N-terminal pro-brain natriuretic peptide
TyG index: triglyceride-glucose index
CMD: coronary microvascular dysfunction
T2DM: type 2 diabetes mellitus
HF: heart failure
AMI: acute myocardial infarction
