# Supplementary material for: Association between the atherogenic index of plasma and adverse long-term prognosis in patients diagnosed with chronic coronary syndrome
Source: Cardiovasc Diabetol. 2023 Sep 21;22:255. doi: 10.1186/s12933-023-01989-z (PMC10515024; doi:10.1186/s12933-023-01989-z)
Supplement: Supplementary file 1 — Supplementary Material 1 [file 12933_2023_1989_MOESM1_ESM.doc]

**Table S1. The characteristics of lipid parameters and antidiabetic drugs in DM patients with CCS**

|  | DM（N=144） |
| --- | --- |
| Lipid parameters |  |
| TC (mmol/L) | 3.81±1.11 |
| HDL-C (mmol/L) | 1.04±0.23 |
| LDL-C (mmol/L) | 2.08±0.93 |
| TG (mmol/L) | 2.10±1.95 |
| AIP | 0.22±0.32 |
| Remnant-C (mmol/L) | 0.69±0.65 |
| Non-HDL (mmol/L) | 2.74±1.10 |
| Antidiabetic drugs |  |
| Insulin, n (%) | 33（22.9） |
| Metformin, n (%) | 60（41.7） |
| Glinides, n (%) | 17（11.8） |
| Sulfonylureas, n (%) | 21（14.6） |
| Thiazolidinediones, n (%) | 6（4.2） |
| DPP-IV inhibitors, n (%) | 4（2.8） |
| Alpha-glucosidase inhibitors, n (%) | 41（28.5） |

*DM* diabetes mellitus*, CCS* chronic coronary syndrome*, TC* total cholesterol, *HDL-C* high-density lipoprotein-cholesterol, *LDL-C* low-density lipoprotein-cholesterol, *TG* triglyceride, *AIP* atherogenic index of plasma, *DPP-IV inhibitors d*ipeptidyl-peptidase-IV (DPP-IV) inhibitors,

**Table S2. Subgroup analysis of the association between the atherogenic index of plasma and MACE.**

|  | **No. of Patients（N=404）** | **Q1（N=101）** | **Q2（N=102）** | **Q3（N=100）** | **Q4（N=101）** | **P value** | HR（95%）CI |
| --- | --- | --- | --- | --- | --- | --- | --- |
| Age |  |  |  |  |  |  |  |
| <65 | 241（59.7） | 48（47.5） | 57（55.9） | 72（72.0） | 64（63.4） | 0.073 | 2.446（0.921-6.495） |
| ≥65 | 163（40.3） | 53（52.5） | 45（44.1） | 28（28.0） | 37（36.6） | 0.143 | 2.007（0.790-5.098） |
| SEX |  |  |  |  |  |  |  |
| male | 238（58.9） | 51（50.5） | 58（56.9） | 63（63.0） | 66（65.3） | 0.074 | 2.151（0.930-4.977） |
| female | 166（41.1） | 50（49.5） | 44（43.1） | 37（37.0） | 35（34.7） | 0.324 | 1.769（0.570-5.492） |
| DM |  |  |  |  |  |  |  |
| Yes | 144（35.6） | 25（24.8） | 38（37.3） | 37（37.0） | 44（43.6） | 0.914 | 0.945（0.339-2.632） |
| No | 260（64.4） | 76（75.2） | 64（62.7） | 63（63.0） | 57（56.4） | 0.009 | 3.730（1.396-9.966） |
| Hypertension |  |  |  |  |  |  |  |
| Yes | 254（62.9） | 57（56.4） | 68（66.7） | 62（62.0） | 67（66.3） | 0.075 | 2.160（0.924-5.047） |
| No | 150（37.1） | 44（43.6） | 34（33.3） | 38（38.0） | 34（33.7） | 0.210 | 2.032（0.671-6.149） |
| LDL-C levels |  |  |  |  |  |  |  |
| <3.4 (mmol/L) | 366（90.6） | 97（96.0） | 92（90.2） | 87（87.0） | 90（89.1） | 0.097 | 1.835（0.896-3.757） |
| ≥3.4 (mmol/L) | 38（9.4） | 4（4.0） | 10（9.8） | 13（13.0） | 11（10.9） | 0.067 | 6.470（0.879-47.633） |
| hsCRP |  |  |  |  |  |  |  |
| <10 (mg/L) | 369（91.3） | 89（88.1） | 94（92.2） | 94（94.0） | 92（91.1） | 0.031 | 2.181（1.073-4.430） |
| ≥10 (mg/L) | 35（8.7） | 12（11.9） | 8（7.8） | 6（6.0） | 9（8.9） | 0.273 | 5.728（0.252-129.967） |

*CCS* chronic coronary syndrome, *DM* diabetes mellitus*, LDL-C* low-density lipoprotein-cholesterol, *hsCRP* High Sensitivity C-reactive Protein

**Table S3. The subgroup analysis of antidiabetic drugs in DM patients with CCS**

|  | **No. of Patients（N=144）** | **AIPquatile** | | | | **P value** | HR（95%）CI |
| --- | --- | --- | --- | --- | --- | --- | --- |
|  |  | **Q1**  **（N=25）** | **Q2（N=38）** | **Q3（N=37）** | **Q4（N=44）** |  |  |
| Insulin |  |  |  |  |  |  |  |
| Yes | 33 | 5 | 6 | 5 | 17 | 0.889 | 0.884（0.158-4.956） |
| No | 111 | 20 | 32 | 32 | 27 | 0.734 | 0.796（0.213-2.972） |
| Metformin |  |  |  |  |  |  |  |
| Yes | 60 | 8 | 11 | 15 | 26 | 0.341 | 2.028（0.473-8.694） |
| No | 84 | 17 | 27 | 22 | 18 | 0.299 | 0.431（0.088-2.115） |
| Glinides |  |  |  |  |  |  |  |
| Yes | 17 | 2 | 6 | 3 | 6 | 0.959 | 0.898（0.015-54.029） |
| No | 127 | 23 | 32 | 34 | 38 | 0.980 | 0.986(0.339-2.870) |
| Sulfonylureas |  |  |  |  |  |  |  |
| Yes | 21 | 5 | 3 | 5 | 8 | 0.766 | 1.452(0.124-17.026) |
| No | 123 | 20 | 35 | 32 | 36 | 0.826 | 0.879(0.280-2.766) |
| Alpha-glucosidase inhibitors |  |  |  |  |  |  |  |
| Yes | 41 | 9 | 12 | 8 | 12 | 0.099 | 0.144(0.014-1.443) |
| No | 103 | 16 | 26 | 29 | 32 | 0.252 | 1.927(0.627-5.921) |

*DM* diabetes mellitus*, CCS* chronic coronary syndrome*, AIP* atherogenic index of plasma

**Table S4. Correlation between the atherogenic index of plasma and other variables in DM patients with CCS**

| Variable | Coefficient | P value |
| --- | --- | --- |
| Age | -0.193 | 0.021 |
| BMI | 0.127 | 0.129 |
| SBP | -0.058 | 0.510 |
| DBP | -0.054 | 0.539 |
| FBG | 0.206 | 0.015 |
| Hb1AC | 0.149 | 0.079 |
| SCr | 0.122 | 0.146 |
| TC | 0.288 | <0.001 |
| HDL-C | -0.640 | <0.001 |
| LDL-C | 0.220 | 0.009 |
| TG | 0.948 | <0.001 |
| LVEF | 0.039 | 0.648 |

*DM* diabetes mellitus*, CCS* chronic coronary syndrome, *BMI* body mass index, *SBP* systolic blood pressure, *DBP* diastolic blood pressure, *FBG* fasting blood glucose, *HbA1c* hemoglobin A1c, *SCr* serum creatine, *TC* total cholesterol, *HDL-C* high-density lipoprotein-cholesterol, *LDL-C* low-density lipoprotein-cholesterol, *TG* triglyceride, *LVEF* left ventricular ejection fraction.

**
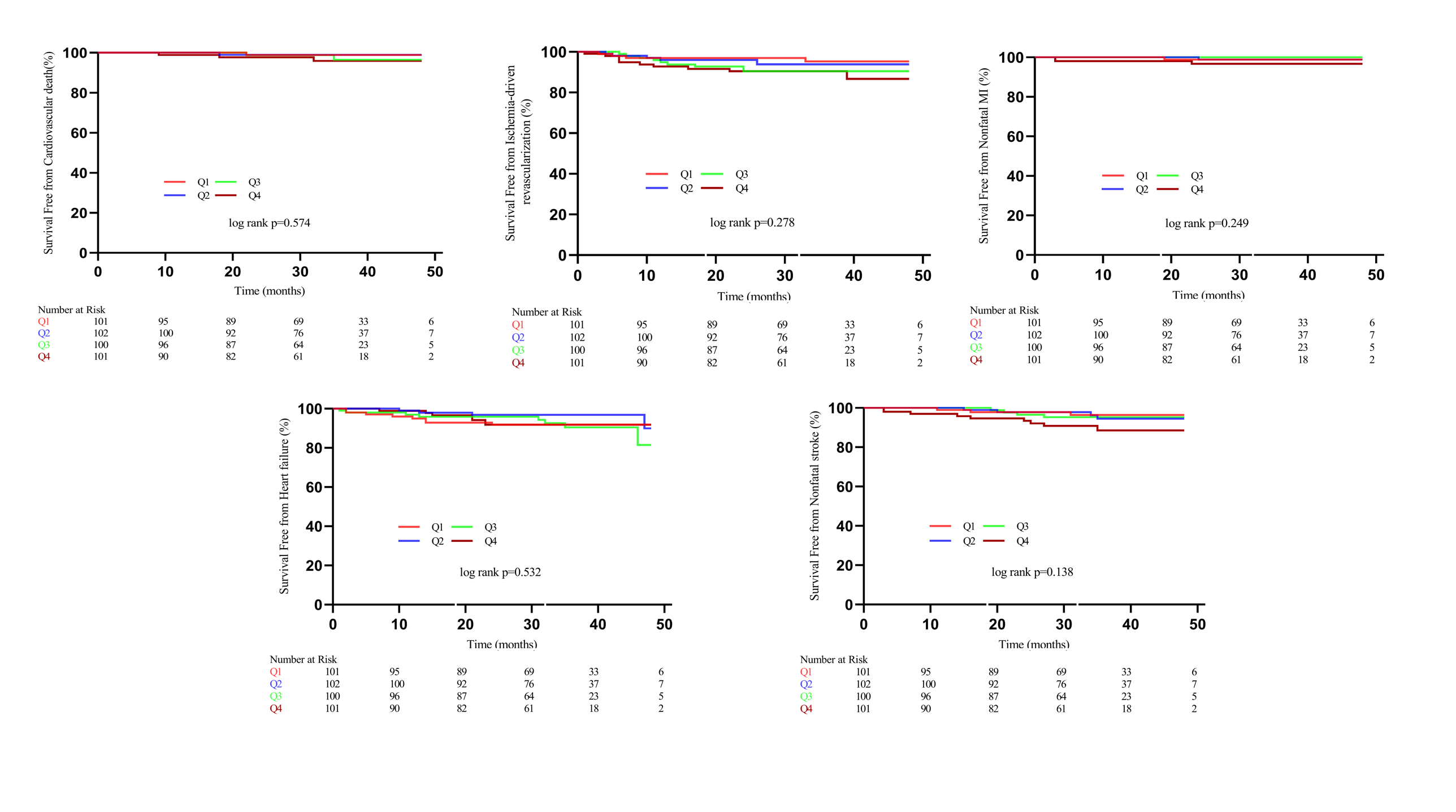
**

Figure S1. Kaplan-Meier survival curve for each component of MACE in patients with CCS.


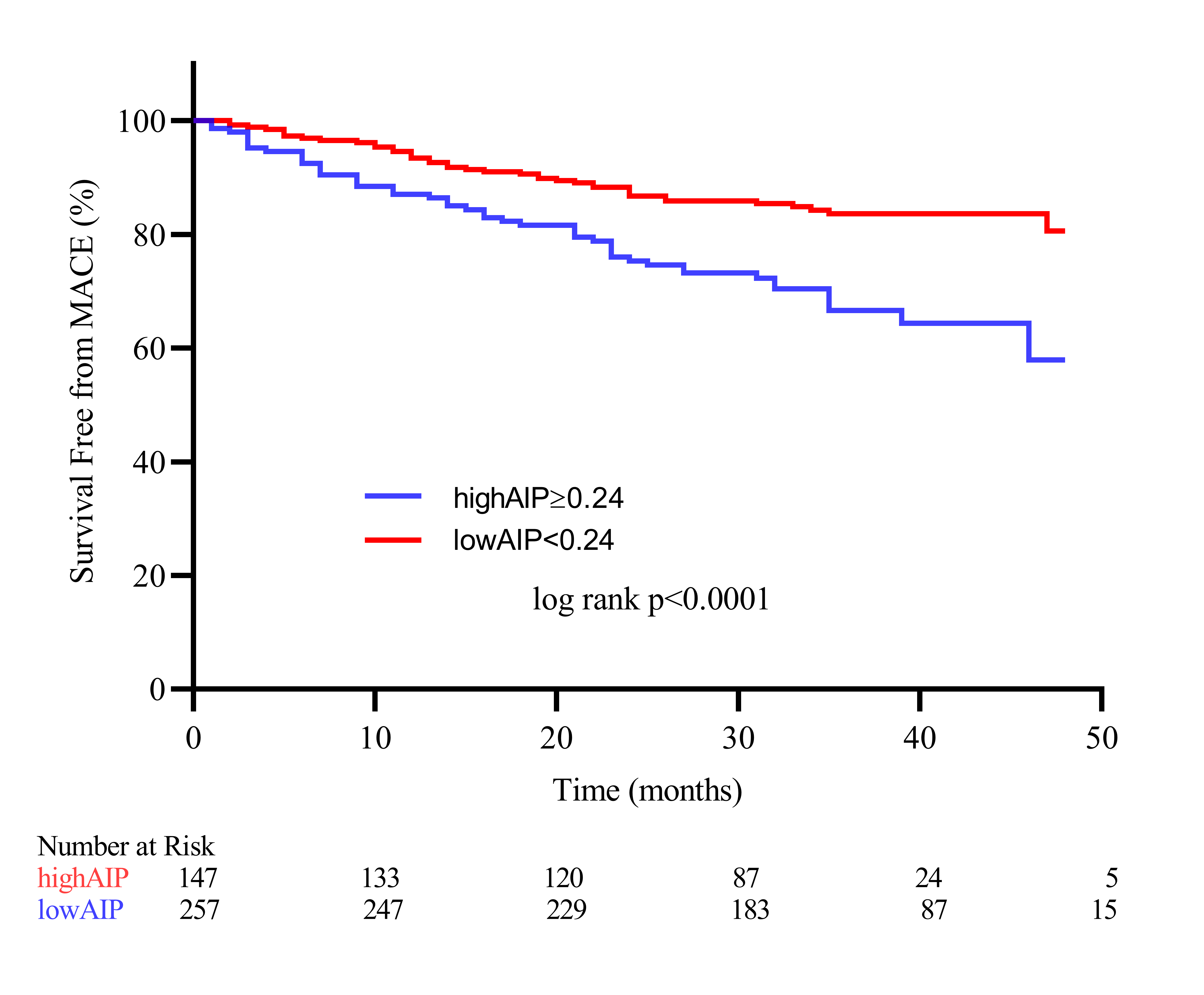
Figure S2. Kaplan–Meier survival curve for MACE in patients with CCS according to cutoff value (0.24).
